# Human blood cell traits and sporadic lymphangioleiomyomatosis: results from mediation joint multi-omics and eQTL Mendelian randomization analysis

**DOI:** 10.1186/s13023-026-04224-6

**Published:** 2026-01-27

**Authors:** Tianshu Liu, Yiting Cai

**Affiliations:** 1 Department of Thoracic Surgery, The Second Affiliated Hospital of Zhejiang University School of Medicine, Hangzhou, China; 2 Department of Reproductive Medicine, The Second Affiliated Hospital of Zhejiang, University School of Medicine, Jiefang Road 88, Hangzhou, 310000 China

**Keywords:** sLAM, Basophils, Mendelian randomization, Immune cells, eQTL

## Abstract

**Background:**

To investigate the genetic causality between Human blood cell (HBC) traits and sporadic lymphangioleiomyomatosis (sLAM) by mediation joint multi-omics and eQTL Mendelian randomization analysis.

**Methods:**

Quality control processes were followed to select eligible instrumental variables strongly associated with 35 kinds of HBC traits. Independent cohort of European ancestry with sLAM and lung function genome-wide association study (GWAS) summary statistics were used separately. We utilized a two-step MR approach to explore potential mediators and evaluate the proportion of effect mediated in the associations linking HBC trait candidates to sLAM. Finally MR analysis integrating single cell expression quantitative trait loci (sc-eQTL) from 14 immune cell types with GWAS of sLAM was conducted.

**Results:**

Increased level of basophil count was positively associated with higher risk of sLAM (BASO#; OR = 3.878, 95%CI:1.137–13.221, *P* = 0.030). No evidences of horizontal pleiotropy were observed. The multivariable MR still demonstrated that BASO# was genetically associated with the risk of sLAM after adjustment for other blood traits in the same category(OR = 5.918, 95% CI:1.275–27.468 *P* = 0.023) and estradiol (OR = 3.814, 95% CI:1.130-12.874, *P* = 0.031) respectively. No evidence for associations between basophil traits (BASO# and basophil percentage of white cells: BASO%) and lung functions containing forced vital capacity (FVC) and forced expiratory volume in 1 s/forced vital capacity(FEV1/FVC). The estimated degree of transitional B cell absolute count mediated the effect of BASO# on sLAM by 36%, while no mediating factors, including immune cells, inflammatory proteins, VEGF-related proteins were found. We identified 12 genes in 14 immune cell types that may have a putative causal relationship with sLAM by mediating through the regulation of BASO#, such as EGFL8, PAX8, KANSL1-AS and L3MBTL3. L3MBTL3 was implicated across several immune cell types.

**Conclusions:**

For the first time, this study leverages mediation analysis and multi-omics MR integrated with sc-eQTL data to elucidate the roles of HBC traits, immune cells, inflammatory proteins, VEGF-related proteins and immune cell-specific genes in the pathogenesis of sLAM among the European populations.

**Supplementary Information:**

The online version contains supplementary material available at 10.1186/s13023-026-04224-6.

## Introduction

Lymphangioleiomyomatosis (LAM) is a multi-system disorder that predominantly affects women of reproductive age. The disorder is characterized by the infiltration of neoplastic smooth muscle-like cells in the lungs [1]. Patients with pulmonary LAM experience symptoms that resemble respiratory failure, such as progressive dyspnea, spontaneous pneumothorax, chylothorax, cough, and chest pain, which are caused by the development of multiple pulmonary cysts replacing the lung parenchyma. LAM remains incurable. Despite the efficacy of existing mechanistic target of rapamycin (mTOR) inhibitor therapy, the disease inevitably relapses once treatment is stopped, ultimately progressing to stages that require lung transplantation. The disease entity is divided in sporadic LAM (sLAM) and LAM associated with tuberous sclerosis complex (TSC-LAM), a rare genetic disorder. This study is restricted to sLAM, because the pathogenic mechanisms and earlier interventions for sLAM have not been completely elucidated.

The pathogenesis of sLAM has been hypothesized to be explained by somatic mutations of the TSC2 gene, although it should be noted that not all sLAM patients exhibit these mutations [2, 3]. There is also a hypothesis suggesting a potential role of female sex hormones in its pathogenesis [3]. Epidemiological observational and in vitro studies have suggested that different cytokines might be involved in sLAM progression, such as vascular endothelial growth factor D (VEGR-D), matrix metalloproteinase and mast-cell tryptase [4, 5]. Human blood cells (HBCs) play a crucial role in various inflammatory and immunological responses occurring in the body. For example, raised circulating counts of eosinophils, the cells with a role in IgE-mediated immunity, are associated with increased asthma risk and asthma-chronic obstructive pulmonary disease overlap risk [6]. Human lung, gastric, colorectal, pancreatic, bladder, prostate cancer, myelodysplastic syndrome and chronic myeloid leukemia have been found to have raised basophils [7–14].Existing literature may suggest that the immune and inflammatory systems regulate the microenvironment and promote tumor progression. However, the association between HBC traits and sLAM (a low-grade, progressive neoplastic disease intrinsically) has not yet been probed.

Mendelian randomisation (MR) is a method in genetic epidemiology that relies on single-nucleotide polymorphisms (SNPs) as instrumental variables(IVs) which are strongly correlated to the exposure of interest to estimate its causal effect on an outcome [15]. By leveraging single-cell expression data, recent genetic studies have cataloged cell-type-specific expression quantitative trait loci (eQTLs), which are genetic variants demonstrating a robust association with gene expression levels in a given cell type [16]. Single-cell eQTL(sc-eQTL) mapping enables the precise identification of causal genes and provides insights into their mechanistic contributions to disease. Methodological innovations in MR now facilitate the assessment of causal mediation pathways, mitigating the biases inherent in traditional observational research.

Here we utilized the most extensive publicly accessible data from genome-wide association study (GWAS) that focused on HBCs and sLAM to assess the causal connection between multiple HBCs and sLAM using a two-sample MR method, mediation and sc-eQTL of immune cells analysis.

## Methods

### Study design

**Fig. 1:**
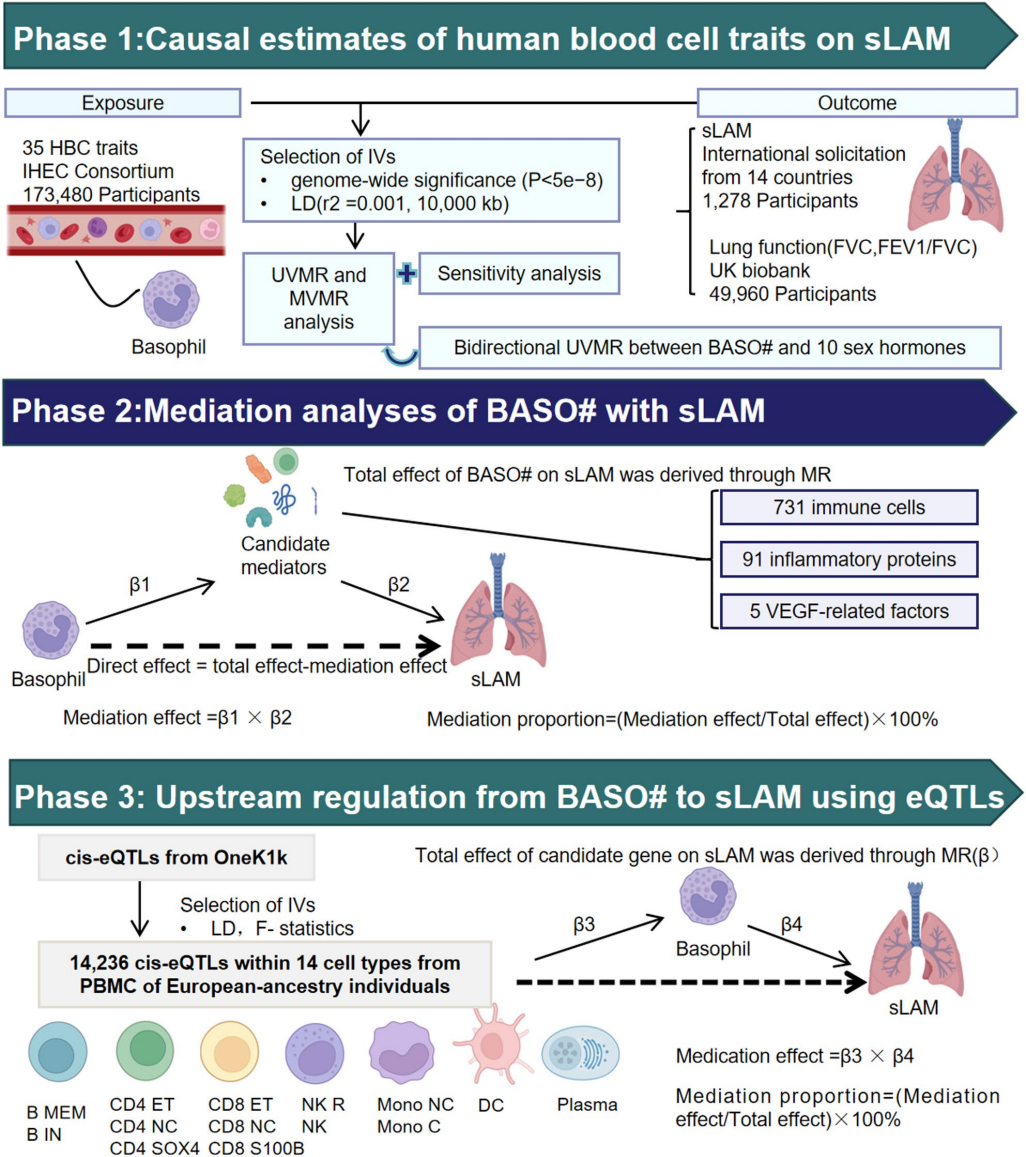
Schematic representation of our design. This MR study consisted of three analysis phases. In phase 1, the causal associations of HBC traits with sLAM, and BASO# trait with lung functions were estimated. MVMR and bidirectional UVMR were both applied reduce the overlapped effects. In phase 2, two-step MR was applied to screen for candidate mediators that may lie in the pathways between BASO# and sLAM. In phase 3, upstream regulation from BASO to sLAM using eQTL and MR. HBC Human blood cell, sLAM sporadic Lymphangioleiomyomatosis, BASO# Basophil count, MR Mendelian randomization; eQTL expression quantitative trait loci, MVMR multivariable MR, UVMR Univariate MR

This MR study consisted of three analysis phases(Fig. 1). In phase 1, two-sample MR analyses were performed to evaluate the causality of 35 HBC traits and sLAM (abbreviations in Table S1). The associations between basophil traits and lung function or sex hormones were assessed respectively, which may exacerbate LAM symptoms and progression. In phase 2, we conducted mediation studies to explore how biomarkers variables mediate the causality between certain HBCs and sLAM, using GWAS data for 91 inflammatory proteins, 731 immune cells and VEGF related factors, the key regulators of LAM. In phase 3, upstream regulation from HBCs to sLAM using sc-eQTL. We explore the upstream regulation from HBCs to sLAM using two-step MR approach. All analyses were performed based on the guidance of STROBE-MR.

### Source of 35 HBC traits

The 35 HBC traits were obtained from a 2016 GWAS study of IHEC Consortium including UK Biobank, UK BiLEVE, and INTERVAL (173480 European-ancestry participants) [17].

### Source of inflammatory protein and VEGF related factor data

The summary data for inflammatory proteins were obtained from a 2023 GWAS on circulating inflammatory proteins, which included 11 cohorts (14,824 European-ancestry participants) and tested 91 inflammatory proteins [18]. Five VEGF related factor data were obtained from a GWAS study of population-based KORA study (1,000 European-ancestry participants) [19]. VEGF-C, VEGF soluble receptor 2 (VEGF sR2), VEGF soluble receptor 3 (VEGF sR3),VEGF and endocrine gland-derived VEGF (EG-VEGF) were employed.

### Source of immune cell data

The immune cell data were obtained from a GWAS study of a Sardinian cohort in the SardiNIA project, encompassing a total of 731 phenotypes:118 absolute cell counts, 192 relative cell counts, 389 surface antigen median fluorescence intensities, and 32 morphological parameters (3,757 participants) [20].

### Source of sex hormone data

Ten sex hormones included oestradiol, oestrogen, estrogen receptor(ER), Anti-Müllerian hormone (AMH), estrogen sulfotransferase (SULT1E1), follicle-stimulating hormone (FSH), luteinizing hormone (LH), sex hormone-binding globulin (SHBG), total testosterone levels (TTL) and bioavailable testosterone levels (BTL). Summary data were obtained through several GWAS studies of European-ancestry(oestradiol:206,927 participants in 2020; oestrogen:337,159 participants; ER, SULT1E1 and LH:3,301 participants in 2018;AMH 7,049 participants; FSH:3,484 participants; SHBG:214,989 participants; BTL:188,507 participants; TTL: 230,454 participant).

### Source of cis-eQTLs of immune cells

We utilized single cell cis-eQTLs of immune cells derived from the OneK1K single-cell eQTL dataset [21]. The QTL mapping data for this eQTL analysis were generated from this resource, which offered high-resolution gene regulatory profiles across 14 immune cell types.

### Source of sLAM data

The sLAM data was acquired from a GWAS study in people of European ancestry [22]. The total sample was 1,278 comprising 426 cases, and 852 controls. Cases were diagnosed based on the International Classification of Disease (ICD) codes.

### Source of lung function data

The lung function data was from a 2021 GWAS study of UK Biobank [23]. Two traits of lung function containing forced vital capacity (FVC) and forced expiratory volume in 1 s/FVC (FEV1/FVC) were analyzed.

### Genetic IV selection and validation

35 HBC traits (showed in Table S1) were selected as the examined factors with obtainable genome-wide significant SNPs. A multi-step filtering strategy was adopted to ensure the validity of the selected IVs. SNPs associated with specific factors at genome-wide significance (*P* < 5e − 08) as candidate IVs were selected for further MR analysis. The SNPs associated with considered inflammatory protein, VEGF related factor and immune cell data in univariable MR(UVMR) analyses were selected at a GWAS level (*P* < 5e − 06, or *P* < 1e − 05) from the GWAS datasets. We retained conditionally independent cis-eQTLs significantly associated with gene expression at a genome-wide significance threshold (*P* < 1e − 5) to minimize false positives. This was determined based on the sample size limitations. SNPs in strong linkage disequilibrium(LD; r2 threshold = 0.001, window size = 10,000 kb) were removed by LD-based clumping to ensure the IVs for each exposure phenotype were independent. The clumping was conducted through the European reference panel of the 1000 Genomes Project, which was used to estimate LD between SNPs. SNPs were discarded with non-concordant alleles and palindromic SNPs with ambiguous strands that could not be corrected when harmonizing the exposure data and outcome data. A threshold of F-statistic larger than 10 was recommended for defining instrument strength to eliminate the weak instrumental variable bias [24].

### Statistical analysis

#### Mendelian randomization

UVMR analyses were performed through different MR methods (inverse variance weighting, IVW; weighted median, WM; weighted mode; simple mode and MR-Egger regression) to estimate the causal associations between 35 HBC traits and sLAM separately. The causal estimates using fixed effects IVW methods by meta-analyzing Wald ratio estimates for each IV were calculated. If significant heterogeneity (*P* < 0.05) was observed, random effects IVW method was chosen. For continuous outcomes, effects are presented as β coefficients with their standard errors, while for binary outcomes, results are expressed as odds ratios (ORs) with 95% confidence intervals (CIs). To minimize false positives, we applied false discovery rate (FDR) correction, and associations with an FDR-adjusted P value < 0.05 were considered statistically significant. To reduce the overlapped effects in the same category of blood traits and from oestradiol, multivariable MR (MVMR) analyses were then conducted using the random effects IVW method. Besides we applied bidirectional UVMR methods to explore causal relationships between HBC trait candidates and sex hormones.

#### Mediation MR analysis

Two-step MR analyses were applied to evaluate the mediating effect of 91 inflammatory proteins, 5 VEGF related factors, 731 immune cell phenotypes on the causal association between genetically determined certain HBCs and sLAM. The first step was to estimate the causal effect of genetically determined HBC trait on each potential mediator using UVMR and the second step was to estimate the causal effect of each potential mediator on sLAM using UVMR. Thereafter the coefficient product method was used to assess the indirect effect of certain HBCs on sLAM through the mediators. The indirect effect was divided by the total effect to derive the proportion of the total effect of the HBC trait on sLAM mediated by the mediator candidate. The estimation of standard errors for the mediation effects was performed using the delta method.

#### Upstream regulation from HBC trait candidate to sLAM using sc-eQTL of immune cells

We employed a two-step MR approach to dissect the direct and indirect effects of immune cell-specific gene expression and HBC trait candidate on sLAM. Among the immune cell-related genes, a varying number of genes were obtained from 14 immune cell types. All SNPs satisfied the criterion of an F-statistic > 10, deemed sufficient to provide the necessary statistical power for MR analysis. First, we investigated the causal relationship between immune cell-specific gene expression and HBC trait candidate. Briefly, the IVW method performs a meta-analysis of SNP-specific Wald ratio estimates (obtained by dividing the SNP-outcome estimate by the SNP-exposure estimate) using a random-effects model, yielding the final causal effect estimate (β3). Subsequently, we estimated the total effect of immune cell-specific gene expression (associated with HBC trait candidate) on sLAM, yielding the final causal estimate β. We then quantified the mediation proportion of HBC trait candidate in the effect of immune cell-specific gene expression on sLAM. The indirect effect, representing the causal effect of immune cell-specific gene expression on sLAM mediated through HBC trait candidate, was estimated using the product of coefficients method (β3 × β4). Consequently, the proportion of the mediated effect was calculated as “indirect effect / total effect” ([β3 × β4] / β).

### Sensitivity analysis

The causal estimates of fixed effects IVW method and MR-Egger regression were both used to detect heterogeneity. The heterogeneity was quantified using Cochran’s Q statistics, and significant heterogeneity was served as significant heterogeneity. MR-Egger regression was performed as sensitivity analyses to control widespread horizontal pleiotropy in MR analyses. A leave-one-out analysis was performed by excluding each SNP in turn and then running MR analysis on the remaining SNPs in order to detect potentially outlying IVs. Our MR analyses were performed under R-4.5.2 software with the package TwoSampleMR, tidyverse, ggplot2, purrr, forestploter, etc.

## Results

### Strength of IVs from SNPs of 35 HBC traits

We systematically curated 60–246 genome-wide SNPs for the 35 HBC traits from the GWAS results through literature searching to examine the associations of these HBC traits with the risk of sLAM (supplementary Table S1, S2 and S17). These factors could be sorted into six groups, including compound white cell (WBC#, EO%, BASO%, NEUT%, MONO% and LYMPH%), myeloid white cell (EO#, EO%GRAN, EO + BASO#, BASO#, BASO%GRAN, BASO + NEUT#, NEUT + EO#), lymphoid white cell (LYMPH#), mature red cell (RBC#, MCV, HCT, MCH, MCHC, HGB and RDW), immature red cell (RET#, RET%, IRF, HLSR% and HLSR#), platelet (PCT, PLT# and MPV). F statistics for each instrument-exposure association was calculated as one parameter for the strength of IVs. The median F statistic was 103.286 (in the range of 65.963-211.212), indicating that those SNPs were strong IVs. Moreover, the SNPs explained 9.22% (in the range of 5.33–24.04%) of the variance in their corresponding HBC traits.

### Causal effects of 35 HBC traits on sLAM

**Fig. 2:**
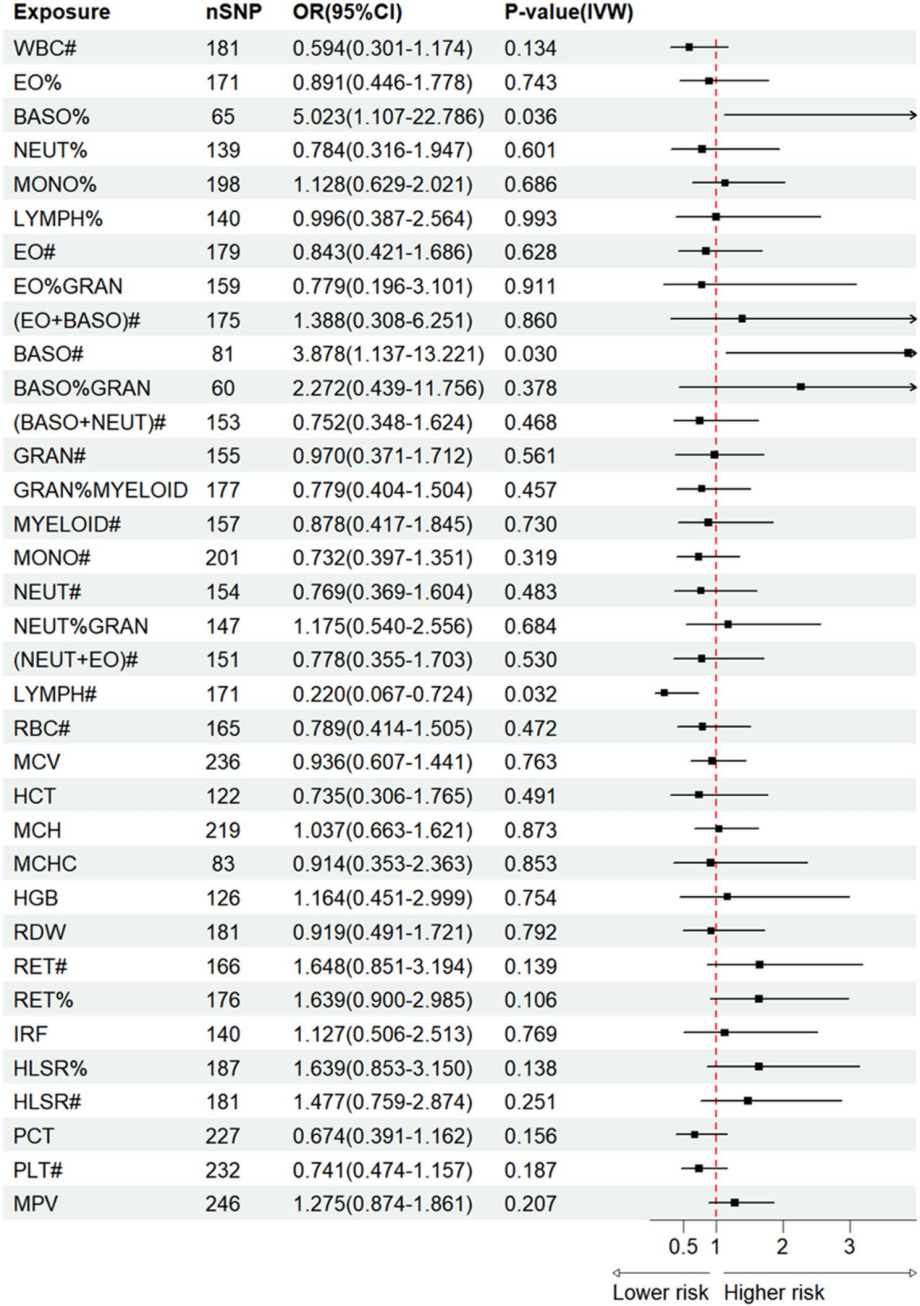
Causal estimates of 35 HBC traits on sLAM by Univariate Mendelian randomization

Primary results of UVMR estimates from different methods are presented in Table S3. Our findings revealed that the genetically predicted BASO% level is causally associated with a increased risk of sLAM (IVW: OR = 5.023, 95%CI: 1.101–22.786, *P* = 0.036; Pheterogeneity = 0.068) (Fig. 2). The random-effect IVW method identified two HBC traits that showed obvious associations with sLAM, including BASO# (IVW random effects: OR = 3.887, 95%CI: 1.137–13.221, *P* = 0.030; WM: OR = 5.505, 95%CI: 1.118–27.115, *P* = 0.036), and LYMPH# (IVWrandom effects: OR 0.220, 95% CI 0.067–0.724, *P* = 0.013; WM: OR = 0.261, 95%CI: 0.075–0.909, *P* = 0.035). Notably, the two basophil traits (BASO% and BASO#) were associated with a raised risk of sLAM (the ORs range from 3.887 to 5.023), while LYMPH# was associated with an reduced risk of sLAM. However, no evidence for associations between other blood cell traits and sLAM was discovered.

**Fig. 3:**
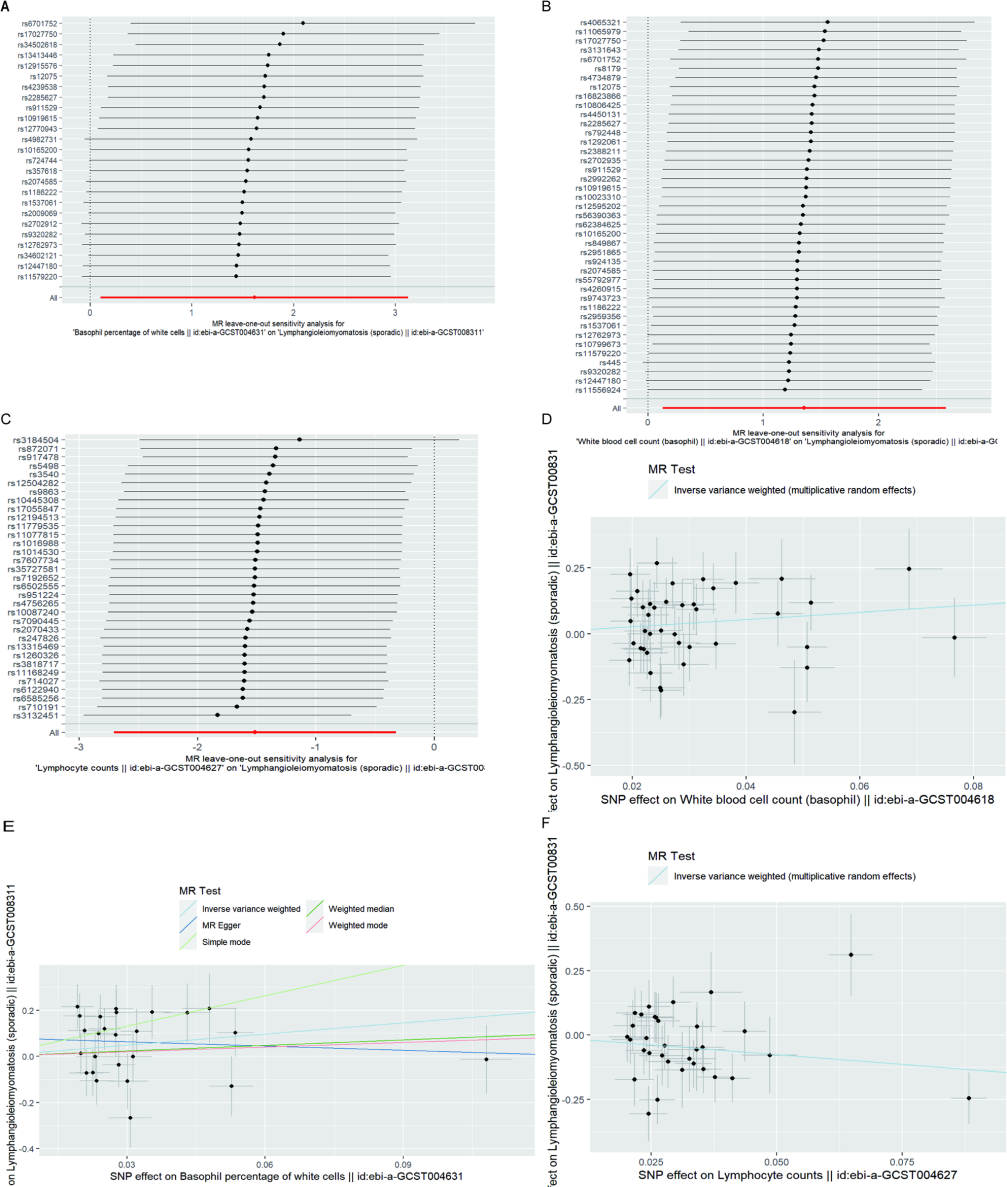
Leave-one-out analyses (**A**-**C**) and Scatter plots (**D**-**F**) of BASO%, BASO# and LYMPH#

In the MR-Egger intercept test, we detected no strong evidences of horizontal pleiotropy (BASO%:Ppleiotropy = 0.975; BASO#:Ppleiotropy = 0.629; LYMPH#:Ppleiotropy = 0.923), indicating robust relationships between the three HBC traits and sLAM (Table S3). As shown above, MR analyses of two HBC traits (BASO# and LYMPH#) associations with sLAM presented some evidences of heterogeneity (*P* < 0.05; Table S3). Leave-one-out analyses and scatter plots were carried out to ascertain potential outliers in the instrumental variable estimation of BASO%, BASO#, and LYMPH# causal effects on the risk of sLAM (Fig. 3;Table S5).

To control for bias introduced by genetic instrument overlaps among different blood cell traits, MVMR analysis was performed (Table S4**)**. After adjustment for EO#, MONO#, LYMPH#, and NEUT#, BASO# still had a potential direct effect on sLAM (OR 5.918, 95% CI 1.275–27.468 *P* = 0.023). Elevated BASO# were causally linked to increased oestradiol and reduced SULT1E1 activity(Table S6; oestradiol:β = 0.007,95%CI:0.001,0.014; *P* = 0.025, Ppleiotropy = 0.325; SULT1E1: IVW:β=-0.202,95%CI:-0.397, -0.008; *P* = 0.041, Ppleiotropy = 0.920), whereas no evidence supported a causal associations of sex hormones with BASO# except for LH (Table S8). Since LH has only one SNP, the genetic correlation between LH and BASO# should be interpreted with caution. Results of relevant leave-one-out analyses were shown in Table S7 and S9. To control for bias introduced by other confounding factors, MVMR still showed the significantly causal association between BASO# and the risk of sLAM (OR = 3.814, 95% CI:1.130-12.874, *P* = 0.031) after oestradiol adjustment (Table S4**)**.

### Causal effects of basophil traits on lung functions

Primary results of MR estimates from different methods are presented in Table S10 -S12. Due to significant observed heterogeneity between basophil traits and lung function traits, random effects IVW was applied. No evidence for associations between basophil traits (BASO# and BASO%) and lung functions (FVC and FEV1/FVC) was discovered (BASO# on FVC: IVW random effects:β = 0.005, 95%CI: -0.031,0.040;*P* = 0.791; BASO# on FEV1/FVC:β=-0.042, 95%CI: -0.093,0.009;*P* = 0.105; BASO% on FVC:β = 0.001, 95%CI: -0.043,0.044;*P* = 0.971; BASO% on FEV1/FVC:β=-0.014, 95%CI: -0.052,0.024;*P* = 0.476). In the MR-Egger intercept test, we detected no strong evidences of horizontal pleiotropy. The results might indicated that there were no significant genetic correlations between basophil traits and lung functions impairment.

### Mediation effects of basophil count on sLAM via different pathways

Firstly the causal effects of BASO# with immune cells were calculate (Table S13). Of 731 immune cells, 38 candidates met screening criteria and were qualified as mediators between BASO# and sLAM. Higher BASO# was causally associated with higher 15 candidates of immune cell traits including transitional B cell absolute count (β:0.295 95%CI:0.023,0.568, *P* = 0.038), ect., as well as lower 23 candidates of immune cells such as CD8 + natural killer T absolute count (β: -0.326, 95%CI: -0.600,-0.051, *P* = 0.020). Of 91 inflammatory proteins, higher BASO# was causally associated with higher C-C motif chemokine 4 levels (β:0.105, 95% CI: 0.006,0.205, *P* = 0.037), hepatocyte growth factor levels (β:0.108, 95% CI: 0.003,0.214, *P* = 0.044), interleukin-18 receptor 1 levels (β:0.117, 95% CI: 0.017,0.217, *P* = 0.022), as well as tumor necrosis factor ligand superfamily member 14 levels (β:0.121, 95% CI: 0.010,0.232, *P* = 0.032). No evidence for associations between other BSAO# and VEGF factors was discovered.

Secondly, MR analysis of immune cells, inflammatory proteins with sLAM was processed separately, we found 22 candidates of immune cells associated with sLAM risks, as well as 3 candidates of inflammatory proteins related to sLAM risks (Table S14). For instance, higher transitional B cell absolute count was causally associated with higher risk of sLAM(OR = 5.220, 95%CI:1.163–23.430, *P* = 0.031). Genetically determined higher Axin-1 levels was positive associated with sLAM risks with ORs (95% CIs) ranging from 6.325(1.310–30.538). T-cell surface glycoprotein CD5 (OR = 0.229, 95%CI: 0.068–0.762, *P* = 0.016) and C-X-C motif chemokine 11 levels (OR = 0.218, 95%CI:0.067–0.713, *P* = 0.012) had protective causal effects on sLAM.

**Fig. 4:**
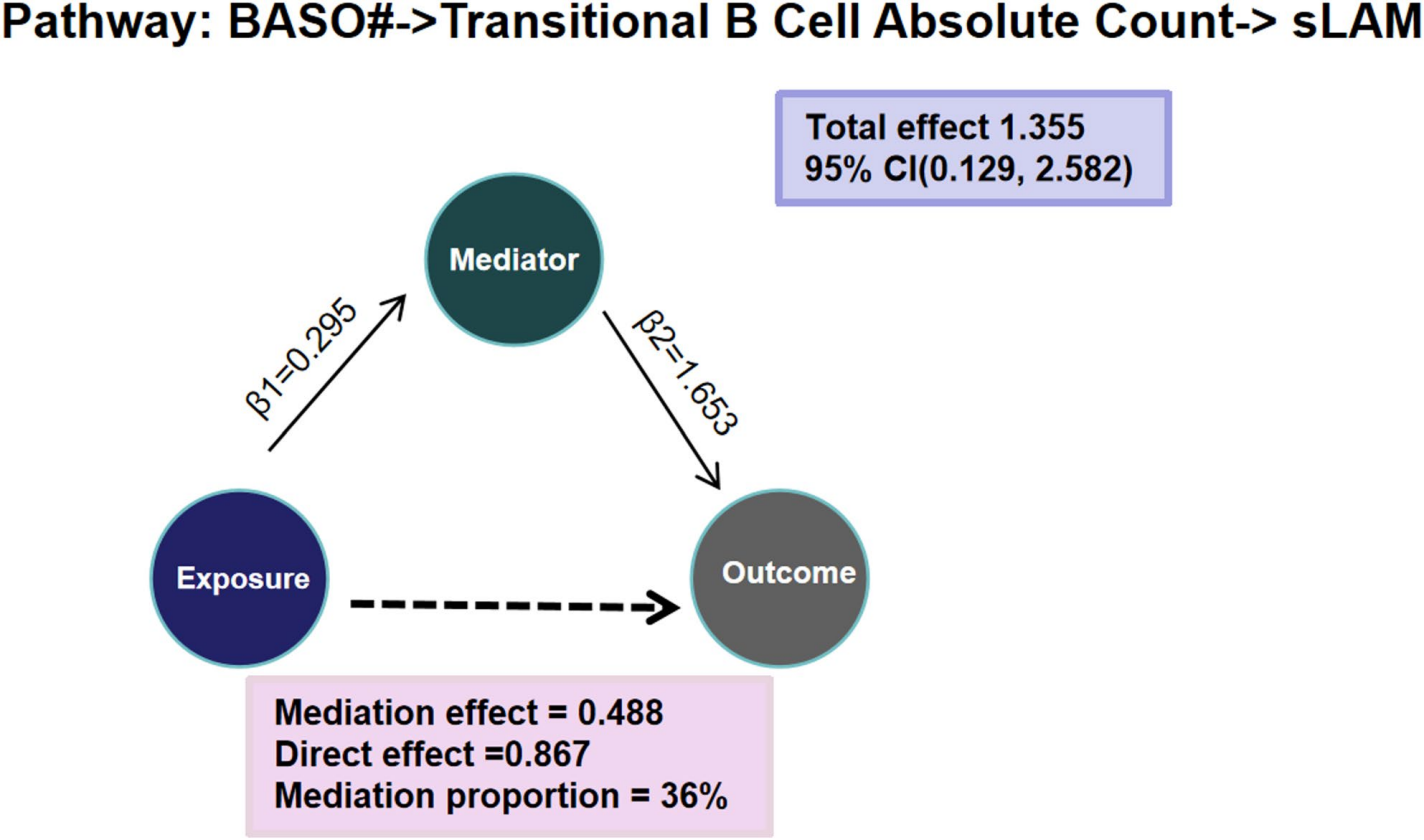
Mediation effects of basophil count on sLAM via immune cells

In brief, we only found that the estimated degree of transitional B cell absolute count mediated the effect of basophil count on sLAM by 36% interestingly(Fig. 4). Sensitivity analysis showed no heterogeneity, horizontal pleiotropy, or outliers in the results except for some data with a limited SNPs (Table S13 and S14).

### Summary of IVs for sc-eQTL of immune cells and explorating upstream regulation from HBC trait candidate to sLAM

**Fig. 5:**
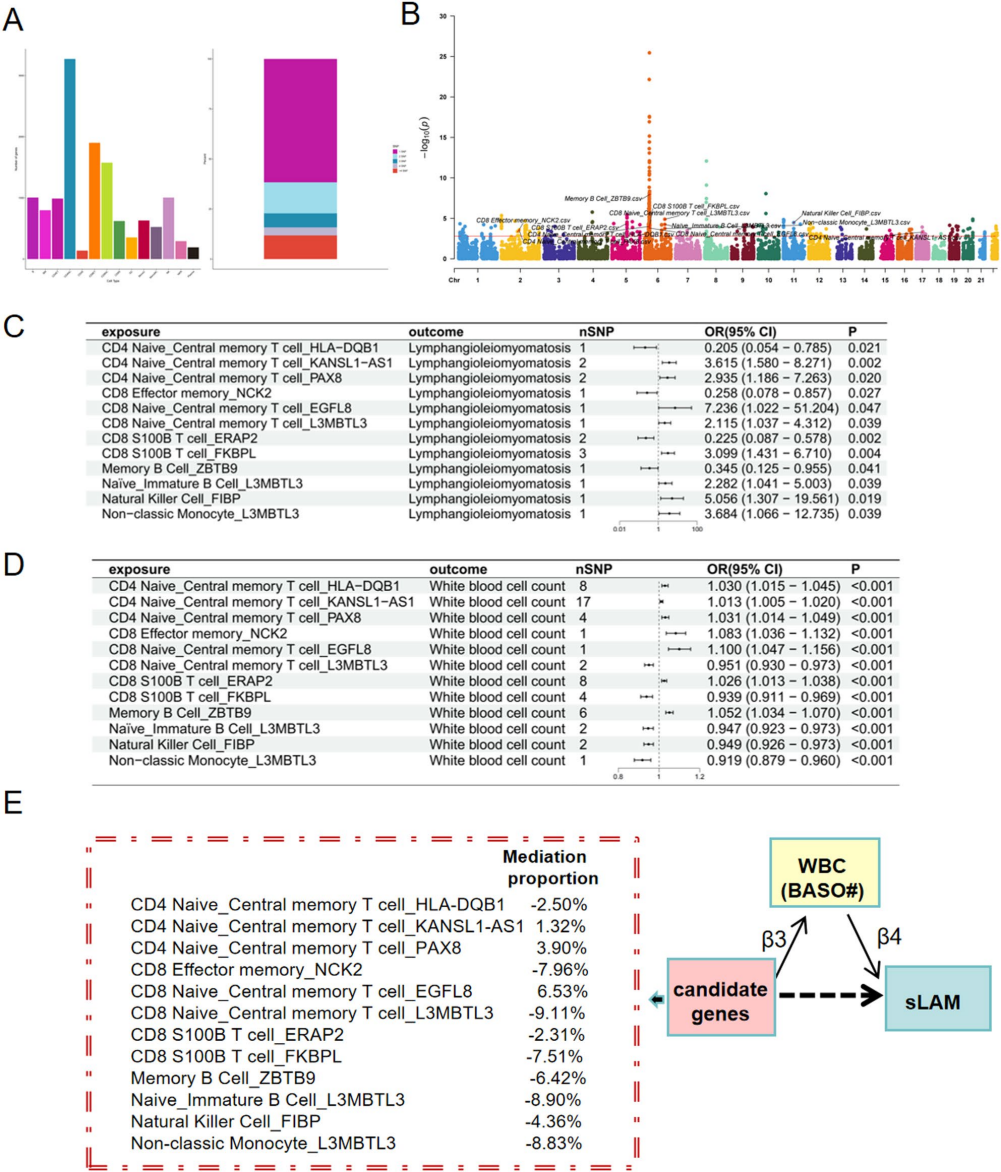
Upstream regulation from basophil count to sLAM using single cell eQTLs. (**A**) Genes identified across 14 immune cell types. (**B**) 393 of positively causal immunogenes. (**C**) and (**D**) The set of 12 genes causally linked to sLAM. (**E**) Mediation effects of genes on sLAM mediated by BASO#

A varying number of genes were identified across 14 immune cell types, most notably in CD4 + naïve conventional T cells and CD8 + effector T cells (Fig. 5A). The majority of SNPs used as instrumental variables consisted of a single SNP, while a minority contained multiple SNPs (Fig. 5A).

As showed in Fig. 2 BASO# promoted the development of sLAM (OR = 3.878, 95%CI: 137-13.221, *P* = 0.030). When assessing the causal effects of immune cell-specific genes on BASO#, 393 genes from different immune cells demonstrated positive causal relationships after adjustment. Examples include memory B cell_ZBTB9 and CD4 naive/central memory T cell_HLA-DQB1, as shown in the P value Manhattan plot (Fig. 5B). Further assessment of the causal relationship between the 393 significant immune cell-specific genes after adjustment and sLAM revealed that 12 out of the 393 genes significantly influenced sLAM (Fig. 5C and D; Table S15 and S16), including CD4 naive_central memory T cell_HLA-DQB1, CD4 naive_central memory T cell_KANSL1-AS1, CD4 naive_central memory T cell_PAX8, CD8 effector memory_NCK2, CD8 naive_central memory T cell_EGFL8, CD8 naive_central memory T cell_L3MBTL3, CD8 S100B T cell_ERAP2, CD8 S100B T cell_FKBPL, memory B cell_ZBTB9, naive_immature B cell_L3MBTL3, natural killer cell_FIBP, and non-classic monocyte_L3MBTL3. Notably L3MBTL3 was implicated across several immune cell types. For instance, deficiency of L3MBTL3 gene expression of naive_immature B cell leading to increased differentiated production of basophils (OR = 0.947, 95% CI:0.923–0.973, *P*<0.001), and an elevated risk of sLAM (OR = 2.282,95% CI:1.104–5.003, *P*<0.001). Further mediation analysis demonstrated that CD8 naive/central memory T Cell_EGFL8 had the highest mediation proportion (6.5%) for its effect on sLAM mediated by BASO#. CD4 naive/central Memory T Cell_KANSL1-AS1 exerted an indirect effect on sLAM mediated by basophil, with a mediation proportion of 1.3%. Similarly, CD4 naive/central Memory T Cell_PAX8 influenced sLAM through BASO# mediation, accounting for a mediation proportion of 3.9% (Fig. 5E and Table S16).These findings suggest that these immune cell-specific genes influence sLAM through the mediation of BASO#.

## Discussion

Based on the findings of this investigation, basophil count may have potential causal effects on sLAM, among which transitional B cell absolute count may serve as a mediator in causality among the European populations. We identified12 genes associated with sLAM through the mediation of basophil counts, involving immune tolerance and regulation of cell differentiation.

Basophils are circulating granulocytes that comprise less than 1% of peripheral blood leukocytes in mammalians, but contributing to a range of inflammatory and immune responses. Interestingly, elevated basophils have been linked to development and recurrence of multiple tumors and to decreased overall survival [7–14]. This study offered some different evidence that higher level of basophils had a deleterious effect on sLAM risk. However, it remains unclear whether raised basophils are associated with the progression of sLAM. Due to extremely limited available GWAS data of sLAM(an orphan disease), stratified analysis of sLAM according to different stage of early, intermediate, advanced, and end-stage are unavailable. A genetic correlation study between basophils and different developmental stages of sLAM could not be conducted. A study has indicated LAM correlated negatively with FVC, and 22 shared genetic variants are uncovered between LAM and pulmonary function [25]. However no significant genetic correlation between basophils and either FVC or the FEV1/FVC deterioration through our MR analysis of basophils on risk of pulmonary function.

Diagnosing sLAM can be challenging, as there is no obvious symptoms at early stage and its symptoms may overlap with other respiratory diseases during progression period. High-resolution computed tomography is highly sensitive and specific for detecting LAM and can reveal the hallmark diagnostic finding of numerous thin-walled pulmonary cysts (≥ 10 cysts with well-defined boundaries). Elevated levels of VEGF-D in the blood, celiac fluid in the thoracic and abdominal cavities, considered tuberous sclerosis, and renal angiomyolipoma or lymphangioma can help confirm the diagnosis of LAM [1, 26]. Finally a lung biopsy may be necessary, which can be performed using transbronchial biopsy or thoracoscopic lung biopsy. Serum marker detection offers considerable advantages in tumor screening in terms of convenience and speed of detection. Current results suggest that screening of basophil count might allow detection of patients with sLAM. It is of signifcance to find some practical, low-cost, and accurate such as basophil count and VEGF-D and then to construct a multivariable model for the early diagnosis of sLAM.

The pathophysiology of LAM is multifactorial and complex, involving numerous interconnected pathways, yet it is not fully elucidated despite recent scientific progress. Somatic TSC2 gene mutations or deletions are found in only a subset of sLAM patients. Mutations in the TSC1/TSC2 genes relieve the inhibition of the mTOR pathway, leading to its hyperactivation and consequent growth, proliferation, and dissemination of LAM cells. The tumor microenvironment is an ecosystem where neoplastic cells interact with recruited immune cells and the supportive tumor stroma [27]. The aberrant immune status in LAM has become a notable focus of recent research. LAM cells share several fundamental hallmarks of cancer, including the expression of CD44/CD44v6 and CD235a that drive tissue invasion and metastasis, as well as the capacity for immune evasion [5, 28]. A subset of LAM cells expresses the transmembrane protein melanoma-associated antigen recognized by T cells. This expression makes these cells recognizable to T cells, thereby increasing their susceptibility to T cell-mediated cytotoxicity [5]. A recent study has shown that macrophages are recruited around LAM cells and induced to polarize toward the M2 phenotype with immunosuppressive functions, thereby driving disease progression [29]. Basophils can also be recruited into tumor microenvironment by several chemotactic molecules produced by tumor and immune cells [30]. We found that 22 immune cell candidates were associated with the risk of sLAM, including B cells, CD4 + T cells, CD8 + T cells, etc.,which was largely consistent with previous studies. However, only transitional B cell absolute count was found to increase the risk of sLAM by mediating basophil count ultimately. Transitional B cells are immature B cells in the periphery, followed by naïve, memory B cells and plasmablasts. The expansion of transitional B cells has been reported in immune diseases including X-linked lymphoproliferative syndrome (a form of common variable immunodeficiency), systemic lupus erythematosus and has also been noted in Immunodeficiency-Centromeric instability-Facial anomalies syndrome and Waldmann’s disease [31]. Previous findings confirm the robust immunomodulatory capacity of CD19^+^CD24^hi^CD38^hi^ transitional B cells [32]. This subset regulates inflammation by suppressing the proliferation and differentiation of CD4 + T cells and CD8 + T cell responses, highlighting its important contribution to maintaining immune tolerance tumor microenvironment. The latest literature demonstrates the existence of a communication pathway between basophils and B cells through exosome release. The spleen (a primary site for B cell maturation and differentiation) and kidneys were the predominant targets of those basophil-derived exosomes. Moreover, the internalization of basophil-derived exosomes by B cells led to a significant improvement in their survival and proliferative capacity [33]. Our speculation suggests that basophil-mediated intercellular communication induces hyperproliferation of transitional B cells, leading to a pro-tolerogenic microenvironment.

We identified 12 upstream regulatory genes linking basophils to sLAM pathogenesis, some of which appear unreported in this pathway. For instance, epidermal growth factor-like domain 8(EGFL8), a negative regulator of immune cell proliferation and activation [34], with the potential to mediate specialized tissue homing, residency, or remodeling of the tissue microenvironment, was identified as a candidate gene. Interestingly, we found that Histone methyl-lysine binding protein 3(L3MBTL3) from naive immature B cell, CD8 naive central memory T cell and non-classic monocyte exhibited a positive effect on sLAM mediated by basophils. L3MBTL3 is a member of the malignant brain tumor family of chromatininteracting transcriptional repressors, which bind histone lysinemethylation H4K20me2 and recruiting other repressive complexes (such as NuRD/CoREST). Knocking out L3MBTL3 in mice leads to the accumulation of immature myeloid progenitors [35]. Reduced expression of L3MBTL33 (rs9375701) is a significant biomarker for breast and prostate cancers [36], Consistent with this, our data indicate that L3MBTL3 acts as a protective factor for basophils. We prudently speculate that dysregulation of L3MBTL3 leads to confusion in the differentiation fate in myeloid and lymphoid lineages in blood cells. Furthermore, cell-cell communication between basophils and transitional B cells contributes to the formation of an immunosuppressive tumor microenvironment, which in turn promotes LAM infiltration and the subsequent development of pulmonary cysts. However, the functions of transitional B cells are still elusive. The causal relationships for these identified genes remain to be rigorously confirmed through functional experiments and replication in larger populations.

Angiogenesis represents one of the hallmarks of cancer. The angiogenic factors, such as the VEGF family and their receptors produced by both cancer cells and infiltrated immune cells, promote tumor angiogenesis [37]. VEGFs can induce basophil chemotaxis through the activation of VEGFR2. VEGF-A acts through the VEGFR-1 and VEGFR-2. VEGF-D activates VEGFR-3 to promote the proliferation of lymphatic endothelial cells and hence promoting lymphangiogenesis. In this study, we did not find a genetic correlation between basophils and VEGF-related factors unfortunately, and VEGFs might not be the mediator through which basophils affect sLAM risk. Estrogen appears to be closely related to LAM, given that evidence from observational studies, containing its predominance in women of childbearing age, the common expression of estrogen and progesterone receptors in lung lesions, and the widely held hypothesis that LAM cells may have a uterine origin. However, paradoxically, estrogen suppression therapy has proven ineffective in controlling LAM progression and may even pose risks, based on results from most available estrogen-specific treatments [3]. Our study indicates a causal effect of elevated basophil count on estradiol levels and estrogen sulfotransferase(an enzyme that catalyzes the transfer of a sulfo group to specific substrates, such as estrogen, regulating their activity and metabolism), whereas reverse MR did not support a causal relationship from estradiol levels or estrogen sulfotransferase to basophil count. This finding is inconsistent with a prior study that reported no causal link between basophils and sex hormones. The discrepancy may be attributed to differences in the cohorts included, as our study expanded the sample size for sex hormones compared to earlier research. Therefore, we further conducted MVMR analysis to account for potential confounding by estradiol.

To our knowledge, this is the first MR study to investigate the association between basophil counts and sLAM using publicly available GWAS summary data. Nonetheless, there are also several limitations. First, our analyses are confined to European-ancestry populations due to the lack of publicly available GWAS data from other ethnic groups. Differences in genetic architecture and environmental exposures across populations may restrict the generalizability of our findings. Future research incorporating data from more diverse ancestries is needed to enhance the external validity of these conclusions. Second, although randomization is designed to minimize confounding, it does not eliminate all individual differences. Uncontrollable variability between participants may still exist in practice, which can affect the comparability of the results.

In conclusion, the MR analysis assessed the causal associations of HBC traits with sLAM among the European populations. Furthermore, it quantified the contribution of immune cell mediation in the basophil-to-sLAM pathway and identified specific immune cell genes that may act as upstream regulators. Basophil-targeting immunotherapy might emerge as viable therapeutic strategies in the future study.

## Supplementary Information

Below is the link to the electronic supplementary material.

Supplementary Material 1

## Data Availability

All the summary-level GWAS data used in the analyses are publicly available. The GWAS data can be obtained through the IEU OpenGWAS database (https://gwas.mrcieu.ac.uk/, OpenGWAS ID: ebi-a-GCST004629 to ebi-a-GCST004633;prot-c-3132_1_1, prot-c-3651_50_5,prot-c-2358_19_2,prot-c-2597_8_3,prot-c-2247_20_11;ebi-a-GCST008311;ebi-a-GCST90025998,ebi-a-GCST90025978;ebi-a-GCST90012105,ukb-b-223;ebi-a-GCST90020092;prot-a-991;prot-a-2892,prot-a-529;ieu-b-4870), the GWAS Catalog (https://www.ebi.ac.uk/gwas/home, accession numbers: GCST90274758 to GCST90274848;GCST0001391 to GCST0002121;GCST90012661;GCST90012102;GCST90012112;GCST90104596), the OneK1K data (https://onek1k.org/), and UKBB (https://pheweb.org/UKB-Neale/,OpenGWAS ID:20003_1140884622) databases.
